# Hypoxia upregulates angiogenesis and synovial cell migration in rheumatoid arthritis

**DOI:** 10.1186/ar2689

**Published:** 2009-05-08

**Authors:** Mohammed A Akhavani, Leigh Madden, Ian Buysschaert, Branavan Sivakumar, Norbert Kang, Ewa M Paleolog

**Affiliations:** 1 Kennedy Institute of Rheumatology, Imperial College Faculty of Medicine, Aspenlea Road, London W6 8LH, UK; 2 Royal Free Hospital, Pond Street, London NW3 2QG, UK; 3 Vesalius Research Center, VIB, Katholieke Universiteit Leuven, Campus Gasthuisberg, Herestraat 49, box 912, 9th floor, 3000 Leuven, Belgium

## Abstract

**Introduction:**

Rheumatoid arthritis (RA) is characterised by invasion of cartilage, bone and tendon by inflamed synovium. Previous studies in our laboratory have shown that hypoxia is a feature of RA synovitis. In the present study, we investigated the consequences of hypoxia on angiogenesis and synovial fibroblast migration in RA.

**Methods:**

Synovial tissue was harvested from RA patients, and synovial membrane cells were cultured under conditions either of hypoxia (1% oxygen) or normoxia (21% oxygen). Protein levels of matrix metalloproteinases (MMPs) and angiogenic factors were measured, while RNA was extracted for PCR quantification of MMPs/tissue inhibitors of MMP (TIMPs) and angiogenic factors. Migration of RA synovial fibroblasts through collagen, and the effect of RA synovial cell supernatants in an *in vitro *angiogenesis assay, were utilised to determine the functional relevance of changes in mRNA/protein.

**Results:**

We observed upregulation under hypoxic conditions of MMPs responsible for collagen breakdown, specifically collagenase MMP-8, and the gelatinases MMP-2 and MMP-9, at both mRNA and protein levels. Increased MT1-MMP mRNA was also observed, but no effect on TIMP-1 or TIMP-2 was detected. RA fibroblast migration across collagen was significantly increased under hypoxic conditions, and was dependent on MMP activity. Furthermore, expression of angiogenic stimuli, such as vascular endothelial growth factor (VEGF), and VEGF/placental growth factor heterodimer, was also increased. Crucially, we show for the first time that hypoxia increased the angiogenic drive of RA cells, as demonstrated by enhanced blood vessel formation in an *in vitro *angiogenesis assay.

**Conclusions:**

Hypoxia may be responsible for rendering RA synovial lining proangiogenic and proinvasive, thus leading to the debilitating features characteristic of RA.

## Introduction

Rheumatoid arthritis (RA) is a chronic systemic inflammatory disorder of unknown aetiology, characterised by altered cellular immunity. Importantly, RA synovium is characterised by an abundance of blood vessels of different sizes [1-4]. Alterations in angiogenic factors, as well as in endothelial cell turnover and apoptosis, have been reported [5-7]. RA is also a disorder in which matrix metalloproteinase (MMP) upregulation ultimately results in destruction of articular cartilage and underlying subchondral bone [8].

The microenvironment of the inflamed joint is characterised by a low partial pressure of oxygen. Low oxygen tension measurements were first recorded in the synovial fluid of patients with RA [9], and subsequent studies demonstrated decreased oxygen tension and glucose levels alongside raised carbon dioxide, lactate and acetate levels, consistent with anaerobic metabolism [10,11]. More recently, our group has confirmed using a sensitive microelectrode technique that synovium in RA patients is more hypoxic than normal synovium [12]. We observed that median synovial oxygen tension in patients with RA was 6% (46 mmHg), compared with 10% (74 mmHg) in patients without RA. Furthermore, we studied patients with RA hand disease, since dorsal wrist swelling due to inflammation of synovium surrounding the tendons of the hand is often the first presentation of RA, and indeed up to 50% of patients with tendon disease can show tenosynovial invasion into the tendon substance itself [13]. We documented that invasive tenosynovium was significantly more hypoxic (median oxygen tension 3%, 26 mmHg) than either noninvasive tenosynovium or joint synovium in the same RA patients, suggesting that hypoxia might be driving invasion of tendon by the synovial tissue, and hence potentially promoting tendon rupture [12]. In the same study, using *in vitro *synovial membrane cell cultures, we demonstrated enhanced secretion of the proangiogenic protein vascular endothelial growth factor (VEGF). While we speculated that this may lead to augmented synovial angiogenesis and/or tendon invasion, however, we were unable at the time to confirm the functional relevance of these findings.

Although the full mechanism for tendon invasion remains unknown, in addition to enhanced angiogenesis, altered expression of MMP and/or the tissue inhibitors of MMP (TIMPs) has been postulated as being responsible for the increased collagen breakdown observed with tendon invasion. The balance between MMP/TIMP is likely to influence cell invasion, in the context of angiogenesis (via degradation of extracellular matrix) and/or in terms of invasion by synovium of underlying tissue such as cartilage, bone and tendon. There is also emerging evidence that MMP may be modulated by alterations in oxygen tension. In endothelial cells, prolonged hypoxia enhanced expression of the gelatinase MMP-2 [14]. Breast cancer cells when cultured in hypoxia showed increased secretion of another gelatinase, MMP-9 [15]. Hypoxia upregulated MMP-2 and MMP-9 activity in a variety of adenocarcinoma cell lines and increased their invasiveness *in vitro *[16]. Crucially, there is evidence that MMPs are regulated by the hypoxia inducible transcription factor (HIF) pathway [17-20]. The role hypoxia plays in regulation of the MMP/TIMP balance in RA, and the *in vivo *relevance of such changes to synovial cell migration, however, have not been investigated.

Previous studies have demonstrated that RA tenosynovial cultures, obtained from patients undergoing wrist extensor tenosynovectomy, produce more MMP-1, MMP-2, MMP-8 and MMP-13 than matched encapsulating tenosynovium [21,22]. RA tenosynovium was subsequently reported more vascular (assessed by measuring CD31 expression) than RA joint synovial lining [23], although the driving force behind such changes remained unclear. Taken together with our demonstration that RA tenosynovium is more hypoxic than noninvasive synovium from the same patients [12], we hypothesised that hypoxia drives angiogenesis and/or synovial invasion. In the present study, we examined the functional relevance of *in vivo *synovial hypoxia in terms of angiogenesis. Furthermore, we examined the effect of hypoxia on MMP/TIMP expression, and the consequences of changes in the MMP/TIMP balance on migration through collagen by RA synovial fibroblasts.

## Materials and methods

### Patient recruitment and tissue culture

A total of 19 patients were recruited at Mount Vernon Hospital, Northwood, Middlesex or at the Royal Free Hospital, Hampstead, London. All patients met American College of Rheumatology 1987 criteria for RA [24]. Full ethical approval was granted for the project (Local Ethics Research Committee EC2003-64). Preoperative informed consent was obtained in all cases.

Operative procedures were carried out under general anaesthetic. Synovial tissue was harvested for the present study from the following procedures: dorsal tenosynovectomy, flexor tenosynovectomy or arthroplasty of the metacarpophalangeal joints. Tissue was collected into DMEM (PAA Laboratories, Coelbe, Germany) containing heat-inactivated 5% FCS (PAA Laboratories) and was digested in DMEM containing 5% FCS, 1 g/l collagenase A (Boehringer Mannheim, Germany) and 0.15 g/l DNAse (Sigma, Poole, UK) [25]. The disaggregated cells were filtered through nylon mesh, and were plated at 1 × 10^6^/ml into 75 cm^2 ^culture flasks (BD Falcon, Leuven, Belgium) under normoxic (21% oxygen) or hypoxic (1% oxygen) conditions using an air-tight hypoxic incubator with inflow and outflow valves (Wolf Laboratories Limited, York, UK). Oxygen concentrations were continuously measured with a built-in oxygen sensor and the percentage of oxygen was adjusted by addition of nitrogen [12,26]. The 3-(4,5-dimethylthiazol-2-yl)-2,5-diphenyltetrazolium bromide colorimetric assay was used to ensure there was no loss of cell viability under hypoxia (data not shown).

After 24 hours of incubation, supernatants were removed and stored at -80°C for protein studies and functional assays, while cellular RNA was extracted as described below.

### Measurement of protein levels for angiogenic factors and MMP/TIMP

Protein concentrations of VEGF/placental growth factor (PlGF) heterodimer, MMP-2, MMP-8, MMP-9 and MMP-13 were measured using commercially available kits (R&D Systems, Abingdon, UK), according to the manufacturer's protocol. To measure VEGF and PlGF, plates were coated with capture antibody for VEGF (1 μg/ml mouse monoclonal anti-human VEGF; R&D Systems) or PlGF (4 μg/ml mouse monoclonal anti-human PlGF; R&D Systems). Anti-human VEGF (200 ng/ml goat polyclonal biotinylated immunoglobulin) and anti-human PlGF (60 ng/ml biotinylated goat polyclonal IgG) detection antibodies were obtained from R&D Systems. Bound PlGF or VEGF was detected using streptavidin-horseradish peroxidise (Amersham Life Sciences, Little Chalfont, UK), followed by 3,3',5,5'-tetramethylbenzidine (Kirkegaard and Perry Laboratories, Gaithersburg, MD, USA). The amounts of VEGF and PlGF were determined in relation to recombinant human VEGF-165 and PlGF protein (R&D Systems).

### Gene expression studies

To measure gene expression, total RNA was isolated using TRIzol™ (Sigma-Aldrich, Poole, UK) followed by phenol/chloroform extraction. To remove any potential DNA contamination, RNA was treated using DNAse with RNasin (Ambion Ltd, Huntingdon, UK). Quantification of the RNA yield from each sample was carried out at 260 nm on a spectrophotometer (Genova, Jenway, Dunmow, UK). cDNA was synthesised using dNTP and Moloney Murine Leukaemia Virus reverse transcriptase (Promega, Southampton, UK).

Exon-spanning PCR primers (MWG, Ebersberg, Germany) for quantitative PCR were designed using Primer 3 Software and UCSC Genome Bioinformatics [26], and are presented in Table 1.

**Table 1:** Exon-spanning PCR primers

	Forward (5' to 3')	Reverse (5' to 3')	Product size (base pairs)
ARP	CTCTGGAGAAACTGCTGCC	TGTAGATGCTGCCATTGTCG	378
VEGF	GTCTTCAAGGAGCGCTGGTTCTG	TAGCCCGGTTCTACCTTCAG	354
PlGF	GAAGCCGGAAGGAGGAGAC	GTCTGTGGCCTTCTCTC	321
MMP-1	GGAGATCATCGGGACAACTC	ACCGGACTTCATATGTCG	529
MMP-2	CAAGTGGTCCGTGTGAAGTATG	CGTCATCGTAGTTGGCTGTG	496
MMP-3	GACAAAGGATACAACAGGGACC	TATCAGAAATGGCTGCATCG	575
MMP-8	GAAGCCGGAAGGAGGAGAC	GTCTGTGGCCTTCTCTC	321
MMP-9	CAAGGATGGGAAGTACTGGCG	TCAACTCACTCCGGGAACTC	464
MMP-13	GATACGTTCTTACAGAAG	GACAAATCATCTTCATCACC	496
MT1-MMP	GTCTTCAAGGAGCGCTGGTTCTG	TAGCCCGGTTCTACCTTCAG	488
TIMP-1	CTTGCTGCTCTACCTCCACC	CTGCATTCACATTTGTTGTGC	548
TIMP-2	GAGTGCAAGATCACGCGCGCTGCC	TGGGAGATCCCTAAGTGCTG	469

ARP, acidic ribosomal protein; MMP, matrix metalloproteinase; PlGF, placental growth factor; TIMP, tissue inhibitor of matrix metalloproteinase; VEGF, vascular endothelial growth factor.

For mRNA quantification, the ABI Prism 7700 Sequence Detection System (Applied Biosystems, Foster City, CA, USA) was used. The following materials were used in PCR reactions: SYBR Green Jumpstart Taq ReadyMix (Sigma), containing dNTP (dATP, dCTP, dGTP, and dTTP), Taq DNA Polymerase, Jumpstart Taq Antibody and SYBR Green I dye. Data were analysed by determining the threshold cycle (Ct) value and were normalised to an endogenous housekeeping gene, acidic ribosomal protein, using the 2-^ΔΔCt ^mathematical model, where ΔΔCt = ΔCt (target sample) - ΔCt (reference sample), and ΔCt is the mean Ct of triplicate reactions of the target gene subtracted from the mean Ct of the housekeeping gene (acidic ribosomal protein). Values were normalised to a reference sample of pooled human cDNA. Validation of the 2-^ΔΔCt ^method was carried out by analysing changes in ΔCt with changes in input cDNA concentration. If the absolute value of the slope was greater than 0.1, primers were re-designed and revalidated.

### Angiogenesis assay

Supernatants from normoxic or hypoxic cultures were centrifugally concentrated at 10,000 × *g *for 90 minutes in Vivaspin 4 spin columns (molecular weight cutoff value of 5 kDa; Sartorius, Epsom, UK) to remove any factors that could confound the subsequent functional assay (such as changes in pH or free radicals). The protein fraction was reconstituted in fresh medium to the same volume as the initial sample. ELISA was performed, as described above, on pre-spun and reconstituted samples, to ensure no loss of VEGF, PlGF or VEGF/PlGF heterodimer. No VEGF, PlGF or VEGF/PlGF protein was detected in the filtrates.

To measure angiogenesis, a commercially available kit was used (AngioKit; TCS Cell Works, Buckingham, UK). Wells were treated on day 0 with or without human recombinant VEGF (2 ng/ml), or in the presence of culture supernatants. Triplicate cultures were examined daily for cell morphology and signs of growth, and medium changes were carried out on days 1, 4, 7, and 9. On day 11, expression of CD31 was visualised by staining with mouse anti-human CD31 antibody (TCS Cell Works), followed by goat anti-mouse IgG alkaline phosphatase and ρ-nitrophenol phosphate, and absorbance at 405 nm was measured. Subsequently, 5-bromo-4-chloro-3-indolyl phosphate/nitroblue tetrazolium (TCS Cell Works) insoluble substrate was added until the tubules developed a dark colour. Wells were washed and air-dried before capture using a BH2 microscope (Olympus Optical, Japan) linked to a KY-F55BE video camera (Victor Company, London, UK).

### Synovial cell migration assay

To prepare rheumatoid fibroblasts, RA synovial membrane cells were isolated as described above. After overnight incubation, nonadherent cells were removed by changing the media and cells were cultured for three passages before use. For the migration assay, porcine collagen type IA (Cellmatrix I-A, 3.0 mg/ml HCl solution, pH 3.0, from porcine tendon; Nitta Gelatin, Osaka, Japan) was neutralised using 1 M NaOH to pH 8.0, and coated onto Boyden chambers (8.0 μm pore size; BD Biosciences, Oxford, UK). A total of 20,000 fibroblasts were added to the top chambers, while bottom chambers were filled with RPMI containing 10% FCS. A universal MMP inhibitor (10 μM *N*-((2R)-2-(hydroxamidocarbonylmethyl)-4-methylpentanoyl)-L-tryptophan methylamide, known as GM6001; Millipore Ltd, Watford, UK) was added to both the bottom and top chambers of selected inserts. Cultures were exposed to either normoxia (21% oxygen) or hypoxia (1% oxygen) for 48 hours. Following migration, inserts were removed and placed in staining solution (CytoSelect Cell Migration assay; Cell Biolabs Inc., San Diego, CA, USA), and then inverted and photographed. Inserts were subsequently placed in extraction solution (CytoSelect Cell Migration assay; Cell Biolabs Inc.). The resulting absorbance was measured spectrophotometrically at 540 nm.

### Statistical analyses

To compare two groups of paired data, Student's two-tailed *t *test was used for normally distributed data and the Wilcoxon signed rank test was used for nonparametric data. For three or more normally distributed groups of data, one-way analysis of variance with Bonferroni's multiple comparison test was used. The GraphPad Prism 5 package was used (La Jolla, CA, USA).

## Results

### Increased synovial membrane angiogenic activity in response to hypoxia

**Figure 1:**
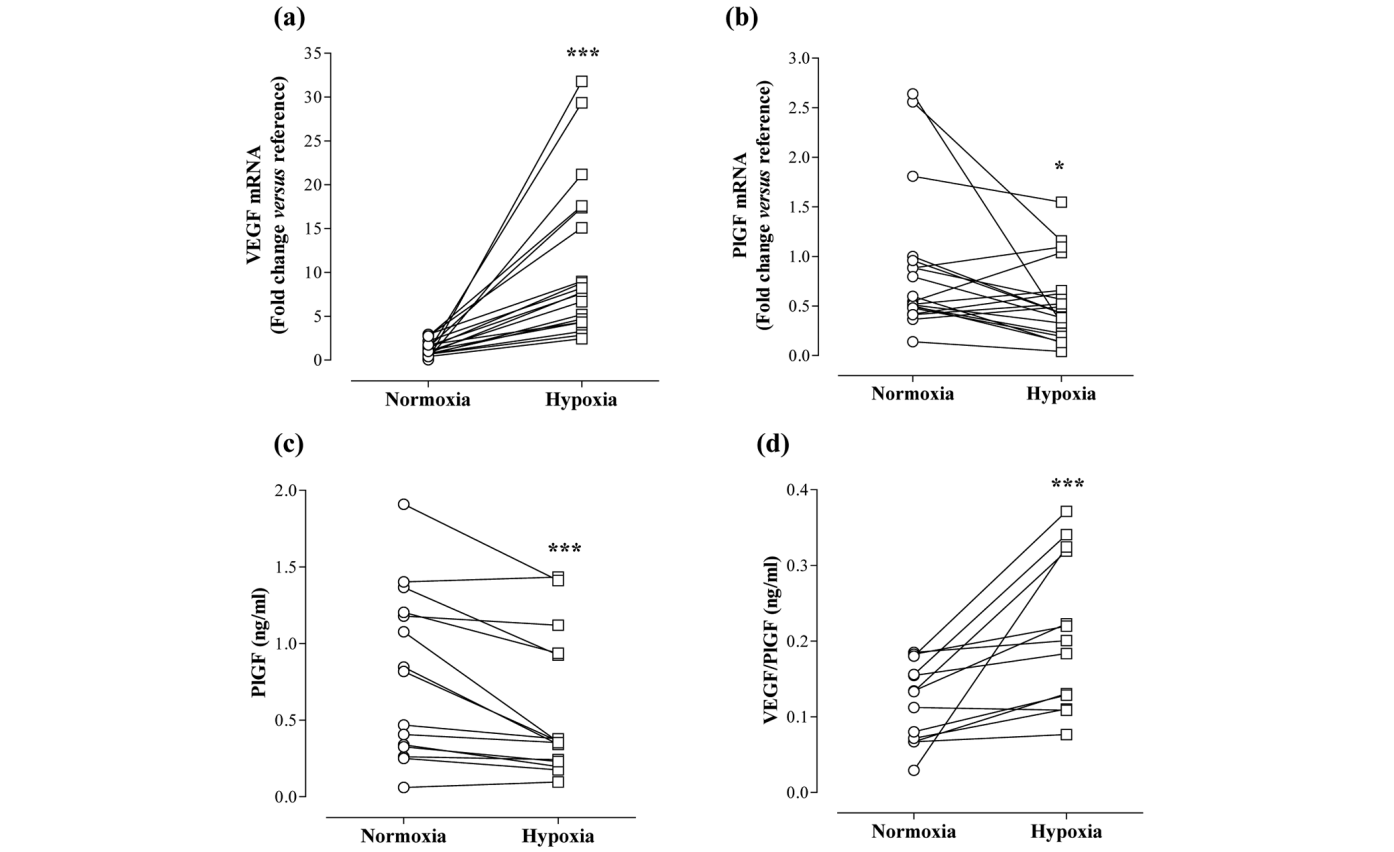
Hypoxia differentially modulates the angiogenic balance. Rheumatoid arthritis synovial cells were exposed to either 21% oxygen (normoxia) or 1% oxygen (hypoxia) for 24 hours. mRNA levels of **(a) **vascular endothelial growth factor (VEGF) and **(b) **placental growth factor (PlGF) were measured by quantitative PCR (n = 19). The change in threshold cycle (ΔCt) values was calculated for each mRNA using the 2-ΔΔ^Ct ^method against the housekeeping gene acidic ribosomal protein. The fold changes in mRNA levels were related to a reference sample (human cDNA). In parallel, release of **(c) **PlGF and **(d) **VEGF/PlGF heterodimer was measured by ELISA of cell culture supernatants (n = 13 to 15). Data were analysed versus normoxia by Wilcoxon signed rank test: *P < 0.05, ***P < 0.001.

We have previously shown that VEGF protein is upregulated by hypoxia in rheumatoid synovial cell cultures [12,27]. To further investigate the effect of hypoxia, parallel cultures were exposed to either 21% oxygen (normoxia) or 1% oxygen (hypoxia) for 24 hours. Our data show that hypoxia significantly increased VEGF mRNA, with a median fold increase of 6.37 relative to matched normoxic cultures (*P *= 0.0001; Figure 1a). In contrast, PlGF mRNA was significantly downregulated by exposure to hypoxia. Median levels of PlGF mRNA following 24 hours of hypoxic culture were 48% of those observed under normoxic conditions, with a decrease observed in 13/19 RA cultures (*P *= 0.0207; Figure 1b). This was confirmed at the protein level, in that hypoxia upregulated release of VEGF protein (data not shown) but reduced levels of PlGF protein in 13/15 culture supernatants (median release: normoxia, 0.82 ng/ml; hypoxia, 0.36 ng/ml; *P *= 0.0006; Figure 1c). Release of PlGF/VEGF heterodimer, however, was increased by hypoxia (median release: normoxia, 0.13 ng/ml; hypoxia, 0.20 ng/ml; *P *= 0.0005; Figure 1d).

We subsequently examined the functional relevance of the upregulation of proangiogenic factors. A total of six patients were studied for this purpose. Following centrifugal concentration, proteins were diluted in fresh medium prior to use in an *in vitro *angiogenesis assay. To ensure that there was no loss of protein through centrifugation of the RA cell culture supernatants, ELISA was carried out on original supernatants, on reconstituted protein fractions and on the aqueous phase remaining after the centrifugation. These tests confirmed that filtration did not significantly affect protein levels of VEGF, PlGF or VEGF/PlGF (Spearman correlation coefficient for comparison of levels in protein fractions versus original fractions = 0.974, *P *< 0.0001; data not shown).

**Figure 2:**
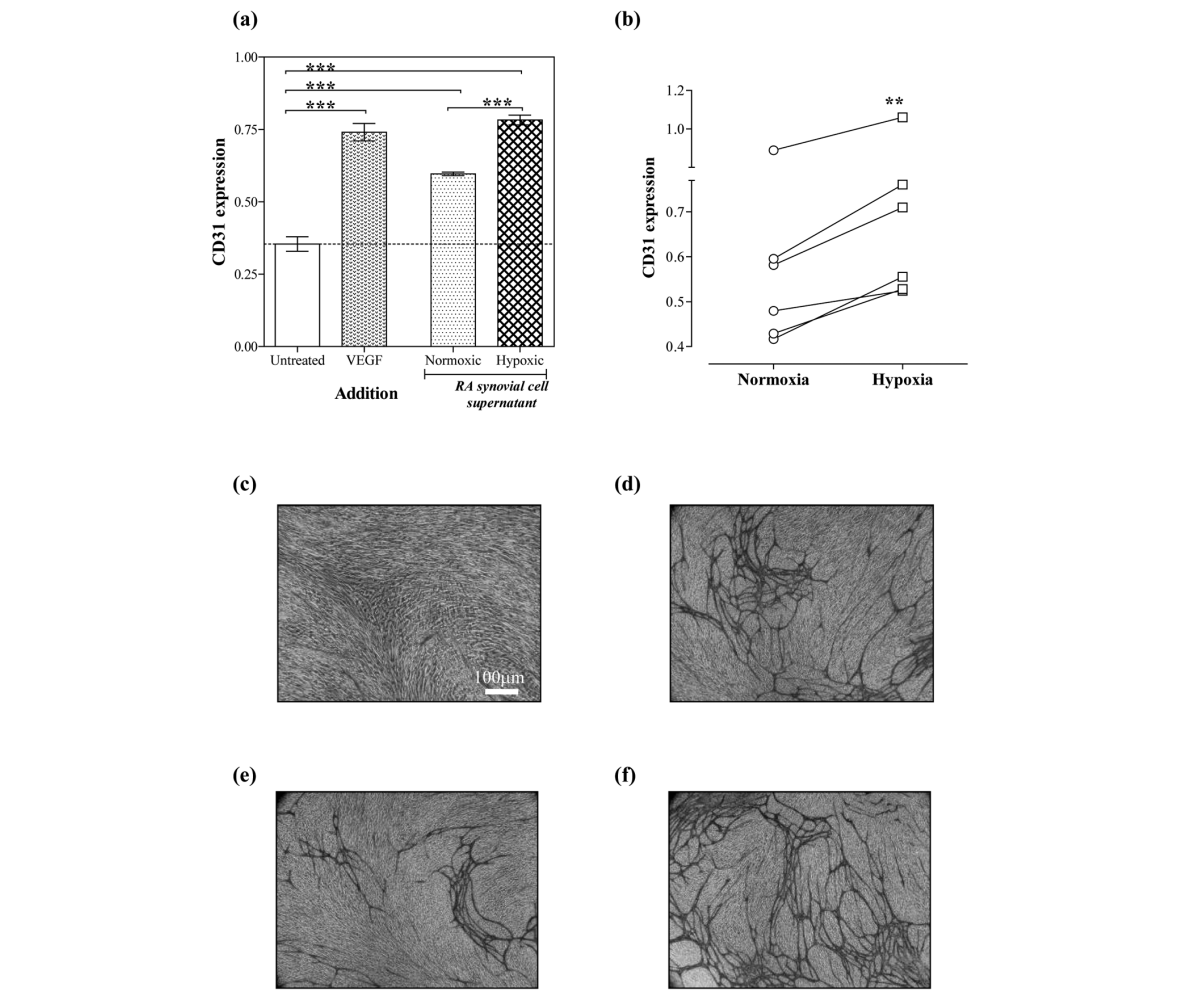
Rheumatoid arthritis synovial cells exposed to hypoxia express more proangiogenic activity. Rheumatoid arthritis (RA) synovial cells were exposed to either 21% oxygen (normoxia) or 1% oxygen (hypoxia) for 24 hours. Cell supernatants were filtered, and the protein fraction was resuspended in fresh medium. Angiogenesis in response to RA synovial cell supernatants was assessed after 11 days, using CD31 expression quantified by colorimetric assay. **(a) **Representative data, with cells exposed to either vascular endothelial growth factor (VEGF) (2 ng/ml), RA synovial cell supernatants or no stimulus. Data are means of triplicate determinations, and were analysed by one-way analysis of variance: ***P < 0.001. **(b) **Comparison of angiogenesis in response to normoxic and hypoxic RA synovial cell supernatants. Data are means of paired triplicate determinations for six separate patients, and were analysed by paired *t*-test: **P < 0.01. **(c) **to **(f) **Representative images showing morphology of the formed tubes stained for CD31 at day 11 (objective magnification, ×40): (c) untreated, (d) VEGF treated (2 ng/ml), (e) normoxic RA synovial cell supernatants and (f) hypoxic RA synovial cell supernatants from the same patient.

Our data show that normoxic synovial cell culture supernatants significantly upregulated angiogenesis *in vitro*. Addition of hypoxic synovial cell culture supernatants, however, upregulated this angiogenic response still further, in comparison with the normoxic supernatants from the same patient. A typical experiment is illustrated in Figure 2a, which shows that VEGF stimulated angiogenesis *in vitro *by 2.09 ± 0.21-fold (mean ± standard deviation), compared with 1.68 ± 0.04-fold and 2.21 ± 0.11-fold for normoxic and hypoxic supernatants, respectively (both *P *< 0.001 versus unstimulated cells, respectively). Furthermore, there was a significant (*P *< 0.001) difference in the angiogenic response induced by hypoxic supernatants relative to normoxic supernatants. These findings were confirmed in synovial cell supernatants from a total of six RA patients, all of which showed greater angiogenic activity for hypoxic supernatants relative to normoxic supernatants (*P *= 0.0013; Figure 2b). Representative images showing the morphology of CD31-positive tubule-like structures obtained in response to either medium alone (Figure 2c), VEGF (Figure 2d), normoxic RA synovial cell supernatants (Figure 2e) or hypoxic RA synovial cell supernatants from the same patient (Figure 2f) are shown.

### Hypoxia enhances synovial cell migration through collagen

**Figure 3:**
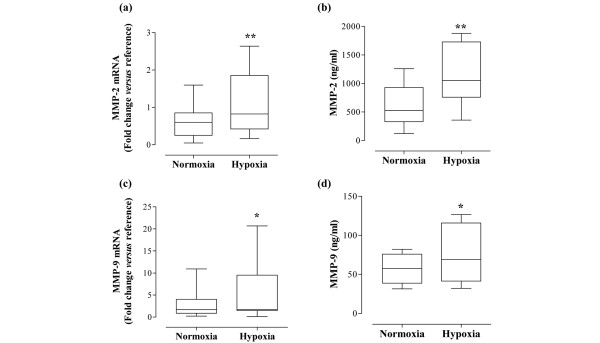
Hypoxia modulates gelatinase expression by rheumatoid arthritis synovial cells. Rheumatoid arthritis synovial cells were exposed to either 21% oxygen (normoxia) or 1% oxygen (hypoxia) for 24 hours. mRNA levels of **(a) **matrix metalloproteinase (MMP)-2 and **(c) **MMP-9 were measured by quantitative PCR (n = 16 to 18). The change in threshold cycle (ΔCt) values was calculated for each mRNA using the 2-ΔΔ^Ct ^method against the housekeeping gene acidic ribosomal protein. The fold changes in mRNA levels were related to a reference sample (human cDNA). In parallel, release of **(b) **MMP-2 and **(d) **MMP-9 protein was measured by ELISA of cell culture supernatants (n = 12). Data were analysed versus normoxia by Wilcoxon signed rank test (b, c) or paired *t *test (a, b) as appropriate: *P < 0.05, **P < 0.01.

In parallel to measuring angiogenic molecules, we examined the effect of hypoxia on MMP/TIMP. Interestingly, in our experiments using RA synovial membrane cells, MMP-2 mRNA levels were significantly upregulated in response to hypoxia, with a median 1.75-fold increase (*P *= 0.006; Figure 3a). The upregulation of MMP-2 mRNA was observed in 16/19 of RA patients. Equally, there was an increase in MMP-2 protein. Median expression of MMP-2 in normoxic conditions was 524 ng/ml (range 122 to 1,259 ng/ml), compared with 1,047 ng/ml (range 358 to 1,877 ng/ml) under hypoxia. The upregulation was observed in 10/12 samples (*P *= 0.0019; Figure 3b). A similar pattern was observed for MMP-9, with mRNA levels upregulated by hypoxia in 11/16 samples by a median of 1.65-fold (*P *= 0.0362; Figure 3c). MMP-9 protein expression in normoxia was in the range 31 to 82 ng/ml (median 57 ng/ml), compared with 32 to 127 ng/ml (median 69 ng/ml) under hypoxic conditions (*P *= 0.0328; Figure 3d).

**Figure 4:**
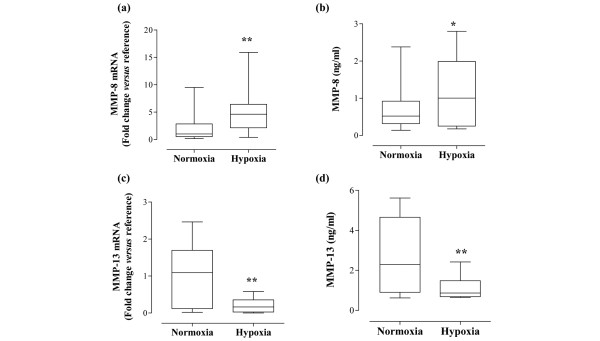
Hypoxia modulates collagenase expression by rheumatoid arthritis synovial cells. Rheumatoid arthritis synovial cells were exposed to either 21% oxygen (normoxia) or 1% oxygen (hypoxia) for 24 hours. mRNA levels of **(a) **matrix metalloproteinase (MMP)-8 and **(c) **MMP-13 were measured by quantitative PCR (n = 10 to 14). The change in threshold cycle (ΔCt) values was calculated for each mRNA using the 2-ΔΔ^Ct ^method against the housekeeping gene acidic ribosomal protein. The fold changes in mRNA levels were related to a reference sample (human cDNA). In parallel, release of **(b) **MMP-8 and **(d) **MMP-13 protein was measured by ELISA of cell culture supernatants (n = 12 to 13). Data were analysed versus normoxia by Wilcoxon signed rank test: *P < 0.05, **P < 0.01.

In terms of collagenase expression, MMP-8 levels were significantly upregulated by hypoxia. There was a 3.09-fold upregulation of MMP-8 mRNA, which was observed in 9/10 patients (*P *= 0.0039; Figure 4a). There was also an increase in MMP-8 protein levels. MMP-8 expression under normoxic conditions ranged from 0.14 to 2.38 ng/ml (median 0.52 ng/ml). For the same samples cultured under hypoxic conditions, MMP-8 levels varied from 0.17 to 2.80 ng/ml (median 1.00 ng/ml, *P *= 0.0266; Figure 4b). In contrast, when cultured under hypoxic conditions, RA synovial cells exhibited significant downregulation of MMP-13 mRNA (*P *= 0.0012; Figure 4c) and protein (*P *= 0.0024; Figure 4d). For example, median MMP-13 protein levels for normoxic cultures were 2.29 ng/ml, compared with 0.87 ng/ml under hypoxia. Overall, 12/14 patients showed decreased MMP-13 mRNA levels, and 10/12 patients showed decreased MMP-13 protein levels when cultured under hypoxic conditions. Levels of another collagenase, MMP-1, were unchanged (Table 2). Furthermore, in our study, MT1-MMP mRNA was upregulated significantly by hypoxia (*P *< 0.01; Table 2), although the increase was relatively modest (1.38-fold, range 0.11-fold to 1.99-fold). Finally, mRNA levels of MMP-3, TIMP-1 and TIMP-2 did not change in response to hypoxia (Table 2).

**Table 2:** Effect of hypoxia on MMP/TIMP mRNA

MMP/TIMP	Normoxia	Hypoxia	*P *value
MMP-1 (n = 19)	0.88 (0.23 to 3.58)	1.11 (0.14 to 6.77)	0.475
MMP-3 (n = 16)	0.38 (0.04 to 1.48)	0.21 (0.01 to 1.09)	0.224
MT1-MMP (n = 19)	1.71 (0.03 to 9.13)	2.45 (0.15 to 10.78)	0.009
TIMP-1 (n = 19)	1.31 (0.03 to 18.90)	2.68 (0.01 to 26.08)	0.131
TIMP-2 (n = 19)	2.57 (0.12 to 20.97)	2.20 (0.24 to 18.64)	0.457

Data presented as the median (range) and were analysed by Wilcoxon signed rank test. Rheumatoid arthritis synovial cells were exposed to either 21% oxygen (normoxia) or 1% oxygen (hypoxia) for 24 hours. mRNA levels of matrix metalloproteinase (MMP)-1, MMP-3, MT1-MMP, tissue inhibitor of matrix metalloproteinase (TIMP)-1 and TIMP-2 were measured by quantitative PCR. The change in threshold cycle (ΔCt) values was calculated using the 2-ΔΔ^Ct ^method against the housekeeping gene acidic ribosomal protein. The fold changes in mRNA levels were related to a reference sample (human cDNA).

**Figure 5:**
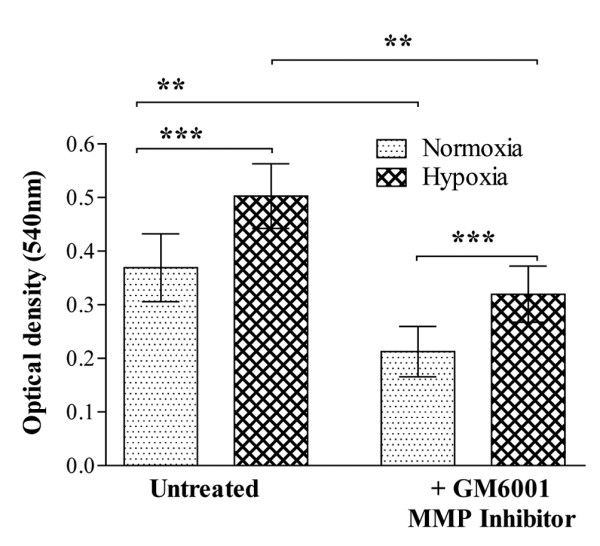
Cell migration through type-I collagen is enhanced under hypoxic conditions: effect of matrix metalloproteinase inhibition. Rheumatoid arthritis fibroblast migration was investigated under normoxic (21% oxygen) and hypoxic (1% oxygen) conditions over a 48-hour culture period. The top chamber was coated with porcine type-I collagen, followed by the addition of 20,000 fibroblasts per well. The chambers were subsequently placed in a 24-well culture plate, with the lower chambers containing DMEM plus 10% FCS. A universal MMP inhibitor (GM6001; 10 μM) was added to both the bottom and top chambers of selected inserts, before exposure to either normoxia or hypoxia. Migration from six different patients, assayed in triplicate, is shown. Data presented as the mean ± standard error of the mean and were analysed by repeated-measures one-way analysis of variance, with Bonferroni's *post hoc *test for multiple comparisons: **P < 0.01, ***P < 0.001.

To study the potential effect of these changes in MMP/TIMP on synovial invasiveness, we utilised a model in which fibroblasts are cultured on a collagen matrix. We confirmed that fibroblasts also upregulate MMP-2 (*P *= 0.0184 by paired *t *test) and MT1-MMP (*P *= 0.0049 by paired *t *test; data not shown). Interestingly, levels of MMP-8 also appeared unaffected by hypoxia, in contrast to the RA synovial membrane cells. To study cell migration, RA fibroblasts were placed in wells coated with type-I collagen and were exposed to either normoxia or hypoxia. We found a significant enhancement of cell migration through collagen under hypoxic conditions, with a median increase in absorbance at 540 nm equivalent to +43% (range +25% to +127%, *P *< 0.001 versus normoxia; Figure 5). This increase in migration was observed for all six patients used, and was evident when the stained cells were studied under the microscope. Interestingly, cell migration was significantly reduced when GM6001, a universal MMP inhibitor, was used. This reduction was observed for both the normoxic conditions (median levels 59% relative to response in the absence of GM6001, *P *< 0.01) and hypoxic cell culture conditions (median levels 58% relative to response in the absence of GM6001, *P *< 0.01). Even in the presence of GM6001, cells incubated under hypoxic conditions exhibited significantly (*P *< 0.001) increased migration when compared with the normoxic GM6001-blocked counterparts (Figure 5).

## Discussion

RA primarily affects the synovial lining, resulting in destructive changes to the joints and soft tissues, most commonly in the hand and wrist. Approximately 50% of patients with RA have tendon involvement, and tenosynovial proliferation can result in tendon adhesions, scarring or rupture [28,29]. The mechanism by which the tenosynovial lining causes tendon damage is poorly understood, but is thought to involve alterations in the MMP/TIMP balance. MMPs play a key role in the degradation of extracellular matrix, as well as in intercellular communication, cell migration, tumour progression and angiogenesis [8,30,31]. Jain and colleagues reported that RA tenosynovial cells produce greater amounts of collagenase (MMP-1, MMP-8 and MMP-13) and gelatinase (MMP-2) enzymes compared with noninvasive tenosynovium [21,22]. Furthermore, our group reported that invasive tenosynovium is more hypoxic than noninvasive RA tenosynovium, or indeed than normal tenosynovium [12]. Intriguingly, hypoxia has been shown to upregulate gelatinases (MMP-2 and MMP-9) [14,15], and to enhance cancer cell invasiveness *in vitro *[16]. MMP-1 and MMP-3 mRNA levels have been reported to be upregulated under hypoxic conditions in RA fibroblasts [32]. Additionally, MMP-13 has been identified as a hypoxia-induced gene in carcinoma cells [33].

The objective of our study was therefore to examine the potential *in vivo *consequences of hypoxia in RA in terms of synovial invasion, by exposing RA synovial membrane cells to 1% oxygen. In our study, hypoxia upregulated MMP-2, MMP-8 and MMP-9, while significantly downregulating MMP-13, at both mRNA and protein levels. This last finding was in keeping with previously reported work, which showed a modest (although not statistically significant) reduction in MMP-13 [23]. We observed no effect of hypoxia on MMP-1 levels, in agreement with published data [23], and no effect on MMP-3, TIMP-1, and TIMP-2. In contrast, MT1-MMP was upregulated at the mRNA level. While there have been previous reports of increased MMP expression/activity in RA fibroblasts exposed to hypoxia, particularly for MMP-1 and MMP-3 [32,34,35], our studies have attempted to mimic RA synovial membrane milieu by utilising total RA synovial membrane cells, which include macrophages as well as fibroblasts, thus possibly explaining the differences between these published data and our own.

To confirm the functional significance of these findings, we investigated the effect of hypoxia on RA synovial cell migration, and observed that hypoxia significantly increased migration of RA fibroblasts through type-I collagen. To assess whether this migration was dependent on MMP activity, we utilised the MMP inhibitor GM6001, which inhibits MMP-1, MMP-2, MMP-3, MMP-8 and MMP-9 [36]. Although migration was significantly reduced by the use of GM6001 in both the normoxic and hypoxic cell cultures, there were still a greater number of cells migrating in the hypoxic cultures, when compared with their normoxic counterparts. This suggests that cell migration through collagen under hypoxic conditions may in part involve MT1-MMP, which is not blocked by GM6001, as well as GM6001-sensitive MMP. Significantly, there is emerging evidence that MT1-MMP is regulated by HIF-2α [17,18]. We and other workers have shown the presence of HIF-1 and HIF-2 in RA synovium [12,37,38], reinforcing the concept that the RA hypoxic milieu may promote alterations in MMP.

As well as affecting matrix degradation, hypoxia is likely to profoundly modulate synovial angiogenesis, through the regulation of angiogenic stimulators such as VEGF [3,4,7]. Co-localisation of HIF-2α and VEGF emphasises the role of hypoxia in the upregulation of angiogenesis in tenosynovitis [12]. We therefore examined the effects of hypoxia on proangiogenic molecules – namely, VEGF, PlGF and VEGF/PlGF heterodimer.

VEGF was used as the control for the present study, as previous studies from our laboratory have consistently shown VEGF protein upregulation in RA synovial cultures under hypoxic conditions [12,23]. In our study, when RA synovial cells were cultured at 1% oxygen, these showed upregulation of VEGF mRNA and protein levels in all samples. Upregulation of VEGF mRNA under hypoxic conditions has been reported previously for temporomandibular joint synoviocytes [39], but we believe this is the first time that VEGF mRNA upregulation in RA metacarpophalangeal synovial and tenosynovial cell cultures has been reported. Furthermore, expression of both PlGF mRNA and PlGF protein was significantly downregulated. Interestingly, PlGF-deficient mice do not display major vascular abnormalities, unlike mice lacking VEGF, suggesting that this molecule is not essential during physiological angiogenesis [40]. PlGF homodimers and PlGF/VEGF heterodimers are present in the synovial fluid of patients with inflammatory arthropathies, including RA [41]. PlGF has previously been reported to be induced by hypoxia in fibroblasts [42]. In contrast, our data showed that PlGF was downregulated by hypoxia, suggesting that hypoxia is probably not the only regulator of PlGF expression in RA.

In cells co-expressing VEGF and PlGF mRNA, VEGF/PlGF heterodimer protein is also expressed, and some of the biological activities attributed to VEGF homodimers may be mediated by VEGF/PlGF heterodimers [43]. Interestingly, our data show that expression of VEGF/PlGF heterodimer in response to hypoxia follows a similar trend to that of VEGF.

We have shown that certain proangiogenic factors are upregulated in RA synovial cell cultures under hypoxic conditions. To investigate whether this translates to an effect on angiogenesis, we used supernatants of the cell cultures from which the mRNA and protein data were obtained, and applied these in an *in vitro *angiogenesis assay. Using this approach, we were able to demonstrate for the first time that hypoxic RA synovial cell cultures induced significantly more vessel outgrowth than their normoxic counterparts, supporting our hypothesis that hypoxia is likely to promote synovial angiogenesis.

## Conclusions

In the present study we have demonstrated that RA synovial cells cultured under hypoxic conditions show upregulation of proangiogenic factors. Crucially, our data also show that hypoxia increases the angiogenic drive of RA cells. Our data additionally provide evidence that certain MMPs are upregulated by hypoxia in RA synovial cells, and that this effect is accompanied by an enhanced capacity of RA cells to migrate through collagen.

Taken together, these data suggest that the hypoxic RA environment promotes and upregulates angiogenesis in inflamed RA synovium, making it proangiogenic and proinvasive. In the context of RA, it seems likely that a disturbance in the balance between MMP/TIMP brought about by tissue hypoxia may determine whether the tenosynovium invades tendons, bone or cartilage.

## Abbreviations

Ct: threshold cycle; DMEM: Dulbecco's modified Eagle's medium; ELISA: enzyme-linked immunosorbent assay; FCS: foetal calf serum; HIF: hypoxia inducible transcription factor; MMP: matrix metalloproteinase; PCR: polymerase chain reaction; PlGF: placental growth factor; RA: rheumatoid arthritis; TIMP: tissue inhibitor of matrix metalloproteinase; VEGF: vascular endothelial growth factor.

## Competing interests

The authors declare that they have no competing interests.

## Authors' contributions

MAA, NK and EMP designed the study. MAA, LM, IB and BS carried out all of the experiments. NK and EMP oversaw the project running and data analysis, and drafted the manuscript. All authors read and approved the final manuscript.
